# Inequity in exercise-based interventions for adults with rheumatoid arthritis: a systematic review

**DOI:** 10.1093/rap/rkac095

**Published:** 2023-01-24

**Authors:** Natalie Jenkins, Nishita Jhundoo, Philippa Rainbow, Katie Jane Sheehan, Lindsay Mary Bearne

**Affiliations:** Department of Population Health, Environmental and Life Course Sciences, King’s College London, London, UK; Department of Population Health, Environmental and Life Course Sciences, King’s College London, London, UK; Department of Population Health, Environmental and Life Course Sciences, King’s College London, London, UK; Department of Population Health, Environmental and Life Course Sciences, King’s College London, London, UK; Department of Population Health, Environmental and Life Course Sciences, King’s College London, London, UK; Population Health Research Institute, St George's, University of London, London, UK

**Keywords:** rheumatoid arthritis, systematic review, exercise, equity factors, interventions, trials

## Abstract

**Objectives:**

This systematic review describes the extent to which PROGRESS-Plus equity factors were considered in the eligibility criteria of trials of exercise interventions for adults with RA.

**Methods:**

Electronic databases were searched for published (Cinahl, Embase, Medline, Physiotherapy Evidence Database), unpublished (Opengrey) and registered ongoing (International Standard Randomized Controlled Trial Number registry) randomized controlled trials (RCTs) of exercise interventions for adults with RA. Two authors independently performed study selection and quality assessment (Cochrane risk of bias tool).

**Results:**

A total of 9696 records were identified. After screening, 50 trials were included. All trials had either some concerns or high risk of bias and reported at least one PROGRESS-Plus equity factor within the eligibility criteria; this included place of residence, personal characteristics (age and disability), language, sex, social capital, time-dependent factors or features of relationship factors. Where reported, this equated to exclusion of 457 of 1337 potential participants (34%) based on equity factors.

**Conclusion:**

This review identified the exclusion of potential participants within exercise-based interventions for people with RA based on equity factors that might affect health-care opportunities and outcomes. This limits the generalizability of results, and yet this evidence is used to inform management and service design. Trials need to optimize participation, particularly for people with cardiovascular conditions, older adults and those with cognitive impairments. Reasons for exclusions need to be justified. Further research needs to address health inequalities to improve treatment accessibility and the generalizability of research findings.

**PROSPERO registration:**

CRD42021260941.

Key messages

- People with RA may not have equal opportunity to participate in exercise trials.
- All included trials excluded potential participants based on at least one equity factor.
- Few studies justified the exclusion of potential participants based on equity factors.

## Introduction

Access to health care is defined as the opportunity or ease with which individuals can access and use the services they need in proportion to their requirements [1]. Guidelines recommend that adults with RA have ongoing access to multidisciplinary team members for rehabilitation and advice. This includes support for and prescription of exercises to improve fitness, enhance the range of movement, strengthen and maintain or restore function. However, access to exercise interventions is highly variable, in part owing to social, environmental and/or health-related factors [2]. Addressing systematic inequities in access to suitable services is a public health priority [3].

Health-care services are commissioned based on evidence of clinical efficacy and cost utility, often from randomized controlled trials (RCTs) or systematic reviews of trials, with or without meta-analyses. However, only a small proportion of people with RA screened for eligibility are reported to take part in exercise trials [4]. It might be that people with similar needs are not equally able to take part owing to factors such as time or financial resources. In contrast, it might be that some people with RA are not invited to participate in studies because they do not meet the eligibility criteria. Systematic exclusion of subgroups of people from trials might lead to the development of exercise interventions that are not suitable for everyone with RA.

This might exacerbate inequities, particularly where those excluded from contributing to the evidence might bear a disproportionate disease burden and might benefit differentially from exercise. Subgroups of people with RA might respond in a different way to exercise interventions owing to differences in equity factors related to social, environmental, physiological or disease states. The PROGRESS-Plus guidance framework {place of residence, race/ethnicity, occupation, gender, religion, education, social capital, socioeconomic status and other factors, such as personal characteristics (e.g. disability), features of relationships and time-dependent relationships [5]} helps to summarize the factors that influence health opportunities and outcomes, such as the chance to participate in exercise interventions [3, 6]. Once subgroups have been identified, a failure to describe them in the baseline characteristics of trial participants or in trial subgroup analyses means that clinicians and decision-makers lack evidence for appropriate management or service commissioning [7]. This might inadvertently perpetuate inequity of access to exercise interventions and health outcomes in adults with RA.

Therefore, the primary objective of this review was to describe the extent to which PROGRESS-Plus equity factors were considered in the eligibility criteria of trials of exercise interventions for adults with RA. Secondary objectives were to describe the extent to which equity factors were considered in baseline characteristics and subgroup analyses in trials of exercise interventions for people with RA.

## Methods

The protocol for this systematic review was registered on the International Prospective Register of Systematic Reviews (PROSPERO: CRD42021260941) [8]. This review was reported in accordance with the preferred reporting items for systematic reviews and meta-analyses equity extension [9].

### Search strategy

Electronic databases were searched from 1 January 2000 to 16 July 2021 for published (Cinahl, Embase, Medline and Physiotherapy Evidence Database), unpublished (Opengrey) and registered ongoing (International Standard Randomized Controlled Trial Number registry) RCTs. The search strategy was based on previously published terms for the population (RA), intervention (exercise) and study design (RCTs) [10, 11] (Supplementary Data S1, available at *Rheumatology Advances in Practice* online).

### Eligibility criteria

This systematic review included RCTs of adults (aged ≥18 years) with an established classification criterion of RA [12–14]. Exercise interventions were defined as a ‘supervised and/or unsupervised programme conducted in an inpatient, outpatient, community, or home‐based setting, including any type of exercise training’ [15]. Multimodal interventions (e.g. exercise and diet) were also included. Eligible study designs included pilot, feasibility or full RCTs. Trials were included irrespective of comparator group or outcome. Non-randomized controlled studies and RCTs published before 1 January 2000 were excluded, meaning that contemporary management of RA was captured [16].

### Study selection

Records were exported and deduplicated in Endnote [17] before being imported into Covidence for screening [18]. Disagreements were resolved by a third reviewer. Two of three reviewers independently screened titles, abstracts and full texts based on the eligibility criteria (Na.J., P.R. and Ni.J.). A third reviewer (L.M.B. or K.J.S.) arbitrated, if necessary.

### Data extraction

Data from included RCTs were extracted by one of three reviewers (Na.J., P.R. or Ni.J.) into a template modified from published extraction templates [19, 20]. Data were checked for accuracy by a third reviewer (L.M.B.). Data included author, year, location, total sample size, eligibility criteria, population, intervention, control, primary and secondary outcome measures, intervention effectiveness for primary outcome and PROGRESS-Plus factors reported in eligibility criteria, baseline characteristics and subgroup analysis. Where available, the number of potential participants excluded based on PROGRESS-Plus factors and justification for exclusion based on PROGRESS-Plus were also extracted.

### Quality assessment

Quality was assessed using the Cochrane risk of bias tool v.2, which enables reviewers to identify bias arising from the randomization process, deviations from the intended interventions, missing outcome data, measurement of the outcome, selection of the reported result and overall bias. Quality assessment was piloted by three reviewers (Na.J., P.R. and Ni.J.) for three RCTs. Uncertainties were resolved by consensus. The remaining RCTs were assessed by one of the three reviewers and checked for accuracy by a fourth reviewer (L.M.B.).

### Data synthesis

Trial characteristics were summarized descriptively. Counts and proportions were used to summarize study characteristics and the extent to which PROGRESS-Plus factors were considered in eligibility criteria, baseline characteristics and subgroup analyses in text, tables and figures. Justifications for exclusion criteria based on PROGRESS-Plus factors were summarized in text, if reported.

## Results

### Study selection

**Figure 1.**
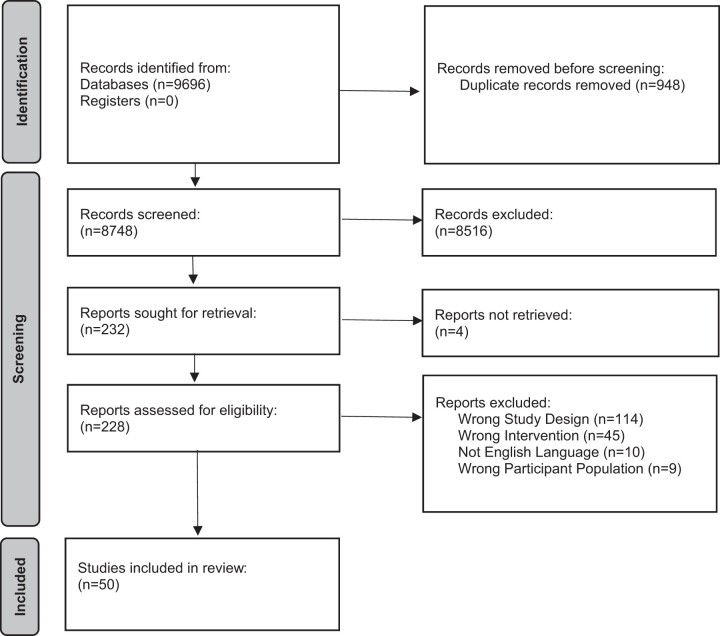
Flow diagram for a systematic review of equity factors in randomized controlled trials of exercise interventions for adults with RA

In total, 8748 records were identified after deduplicate. A total of 228 full texts were screened. Fifty studies met the eligibility criteria and were included in this systematic review (Fig. 1).

### Study characteristics

Overall, this review included 48 full trials [21–68], one feasibility trial [69] and one pilot trial [70]. A total of 4382 participants were included (sample size ranged from 20 [66] to 490 [46] participants). Participant ages ranged from 18 [27, 32, 33, 36, 38, 45, 55, 67, 70] to 87 years [22]. The majority of participants were female (*n* = 3431) [21–70].

Interventions included strengthening exercise (*n* = 26) [21, 23–25, 27, 30, 31, 35, 36, 38, 42, 43, 45–48, 50, 51, 54, 55, 58, 59, 61, 63, 64, 68], aerobic exercise (*n* = 17) [21, 23–25, 27, 29, 36, 38, 44, 45, 47, 55, 59, 61, 63, 64, 68], flexibility exercises (*n* = 10) [25, 30, 31, 35, 36, 46, 51, 54, 63, 64], yoga (*n* = 8) [37, 39–41, 52, 56, 62, 67], walking (*n* = 5) [30, 36, 57, 69, 70], hydrotherapy (*n* = 4) [26, 27, 33, 60], proprioception (*n* = 3) [25, 28, 51], tai chi (*n* = 1) [66] and non-specified exercise-based interventions (*n* = 6) [22, 32, 34, 49, 53, 65]. Comparators included usual care (*n* = 22) [22, 27–29, 32, 34, 36–41, 45, 46, 50, 52, 53, 57, 58, 62, 64, 68], an alternative exercise intervention (*n* = 18) [21, 25, 26, 31, 33, 35, 42–44, 47–49, 51, 59–61, 63, 67], education and advice (*n* = 10) [23, 24, 30, 54–56, 65, 66, 69, 70] or diet (*n* = 2) [38, 55].

### Quality appraisal

Thirty-five studies were considered to be at high risk of bias [21–24, 26, 29, 31, 34–43, 45, 48, 49, 51–53, 55, 57–61, 63–67, 69]. The most common reason for high bias assignment was the selection of the reported results (*n* = 17) [22, 26, 29, 31, 35–43, 48, 49, 53, 69]. Fifteen studies had an overall judgement of some concerns [25, 27, 28, 30, 32, 33, 44, 46, 47, 50, 54, 56, 62, 68, 70], and no studies were deemed low risk of bias (Supplementary Table S1, available at *Rheumatology Advances in Practice* online).

### Synthesis

#### Eligibility criteria

**Figure 2.**
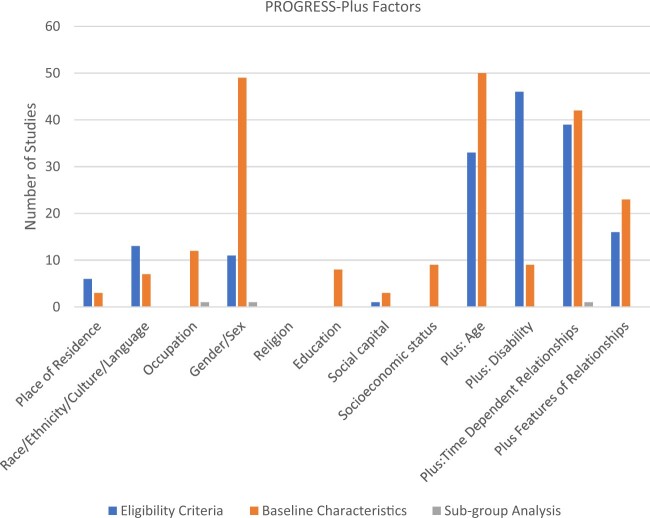
Reporting of PROGRESS-Plus factors in eligibility criteria, baseline characteristics and subgroup analysis

At least one PROGRESS-Plus factor contributed to eligibility criteria in all 50 studies (Fig. 2; Tables 1 and 2). PROGRESS-Plus factors reported in the eligibility criteria included: place of residence (*n* = 6) [21, 22, 30, 43, 53, 59], race/ethnicity/culture/language (*n* = 13) [21, 24, 30–34, 47, 50, 59, 66, 67, 69], gender/sex (*n* = 11) [22, 31, 38, 55, 57, 58, 60, 64, 65, 67, 68], social capital (*n* = 1) [57], plus factor age (*n* = 33%) [21, 23, 24, 26–29, 31–41, 44, 47, 50–52, 55–58, 60–62, 64–66], plus factor disability (*n* = 46) [21–31, 33–41, 44–48, 50–70], plus factor time dependent (*n* = 39) [21, 23, 25, 26, 28, 31, 33–42, 44–49, 51, 52, 54–60, 62–64, 66–70] and plus factor features of relationship (*n* = 16) [29, 35, 37–42, 48, 55–57, 59, 60, 62, 64]. Occupation, religion, education and socioeconomic status did not contribute to eligibility criteria.

**Table 1.** PROGRESS-Plus factors reported in eligibility criteria, baseline characteristics and subgroup analysis

	Eligibility criteria [*n* (%)]	Baseline characteristics [*n* (%)]	Subgroup analysis [*n* (%)]
PROGRESS			
Place of residence	6 (12) [21, 22, 30, 43, 53, 59]	3 (6) [57, 59, 67]	–
Race/ethnicity/culture/language	13 (26) [21, 24, 30–34, 47, 50, 59, 66, 67, 69]	7 (14) [44, 46, 50, 52, 66–68]	–
Occupation	–	12 (24) [22, 23, 26, 34, 43, 45, 46, 50, 53, 57, 67, 70]	1 (2) [43]
Gender/sex	11 (22) [22, 31, 38, 55, 57, 58, 60, 64, 65, 67, 68]	49 (98) [21–24, 26–70]	1 (2) [21]
Religion	–	–	–
Education	–	8 (16) [22–24, 45, 52, 57, 67, 70]	–
Social capital	1 (2) [57]	3 (6) [45, 49, 62]	–
Socioeconomic status		9 (18) [22, 23, 26, 34, 39, 47, 49, 62, 63]	–
Plus: personal characteristics			
Age	33 (66) [21, 23, 24, 26–29, 31–41, 44, 47, 50–52, 55–58, 60–62, 64–66]	50 (100) [21–70]	–
Disability	46 (92) [21–31, 33–41, 44–48, 50–70]	9 (18) [21, 23, 38, 47, 55, 58, 59, 63, 65]	–
Plus: time-dependent relationships			
Disease duration, years/months	10 (20) [21, 25, 26, 34, 35, 37, 42, 47, 59, 69]	42 (84) [21, 23, 24, 26–36, 38, 40–54, 56–58, 60, 61, 63–69]	1 (2) [46]
Previous/upcoming surgery/joint injection	21 (42) [21, 23, 31, 33, 35, 39–41, 44, 45, 47, 48, 51, 52, 54, 56, 59, 60, 62, 67, 68]	–	–
Duration of medication	12 (24) [23, 26, 31, 33–36, 45, 46, 54, 58, 68]	1 (2) [36]	1 (2) [46]
Current exercise participation	21 (42) [28, 33, 36–40, 45, 47–49, 52, 55–57, 59, 62–64, 66, 70]	2 (4) [44, 67]	–
Plus: features of relationships			
Type of medication/supplements	15 (30) [35, 37–42, 48, 55–57, 59, 60, 62, 64]	23 (46) [21, 23, 26, 27, 29, 30, 32, 33, 35, 38, 42, 44, 46, 47, 49, 56, 58, 60, 63, 66–68, 70]	–
Living alone	1 (2) [29]	–	–

**Table 2.** PROGRESS-Plus disability factors reported within the eligibility criteria, baseline characteristics and subgroup analysis

Disability factor	Eligibility criteria [*n* (%)]	Baseline characteristics [*n* (%)]
Smoking status	1 (2) [64]	8 (16) [21, 23, 30, 42, 47, 58, 59, 63]
History of alcoholism or drug abuse	1 (2) [37]	–
Contraindications to exercise	13 (26) [23, 26, 27, 31, 33, 38, 41, 48, 51, 60, 65, 68, 69]	–
Mobility limitations	10 (20) [23, 28, 30, 31, 35, 52, 55, 56, 60, 70]	–
Auditory or visual deficits	1 (2) [60]	–
Poor skin integrity	1 (2) [60]	–
Frailty	1 (2) [22]	–
Falls risk	1 (2) [30]	–
Incontinence	2 (4) [33, 60]	–
RA disease severity	4 (8) [27, 45, 58, 70]	–
Limb loss	3 (6) [29, 53, 60]	–
Pregnancy	2 (4) [23, 33]	–
Co-morbidities		
Cardiovascular conditions, total	39 (78)	10 (26)
Chronic/congestive heart failure	4 (8) [30, 38, 55, 56]	–
Cardiac arrhythmia	3 (6) [21, 28, 61]	–
Myocardial infarction	3 (6) [23, 59, 61]	–
Ischaemic heart disease	3 (6) [28, 47, 56]	–
Thoracic/chest pain	3 (6) [28, 30, 59]	–
Cardiovascular disease	9 (18) [21, 36, 44, 53, 58, 63, 64, 66, 68]	3 (6) [21, 47, 65]
Circulatory problems	1 (2) [60]	–
Cardiovascular risk factors	6 (12) [25, 28, 33, 37, 59, 61]	4 (8) [38, 55, 58, 59]
Respiratory/lung diseases	7 (14) [28, 30, 44, 53, 56, 59, 64]	2 (4) [21, 47]
Neuromuscular disorders	1 (2) [37]	–
Autoimmune disorders	8 (16) [37–41, 48, 55, 62]	–
Musculoskeletal conditions	6 (12) [33, 35, 44, 50, 54, 58]	1 (2) [58]
Malignancy	6 (12) [23, 30, 38, 48, 56, 61]	1 (2) [21]
Neurological disorders	7 (14) [23, 33, 51, 54, 60, 61, 64]	–
Kidney/liver disease	4 (8) [38, 55, 60, 64]	–
Diabetes mellitus	5 (10) [25, 28, 33, 37, 64]	4 (8) [38, 47, 55, 58]
Other chronic or acute co-morbidities	3 (6) [57, 60, 67]	1 (2) [23]
Thyroid disease	4 (8) [28, 30, 55, 56]	3 (6) [38, 47, 55]
Non-specified co-morbidities	1 (2) [34]	2 (4) [47, 63]
Other inflammatory conditions	1 (2) [52]	–
Reproductive diseases	1 (2) [57]	–
Serious mental health conditions	7 (14) [22–24, 48, 51, 60, 69]	1 (2) [23]

#### Justification for eligibility criteria

Nineteen studies included justification for at least one eligibility criterion [21–23, 30, 33, 38, 39, 44, 47, 50, 53, 55, 57, 58, 60–62, 68, 69]. One trial excluded potential participants based on language owing to the financial costs of a translator [69]. One trial excluded participants owing to the potential influence that sex [55] might have on the outcome measure. Three studies provided justification for excluding participants owing to age [21, 57, 58].

Fourteen studies excluded participants based on disability. Where provided, the justification for excluding people with disabilities were as follows: unable to participate in the intervention owing to safety (e.g. contraindication, infection control, cognitive impairment; *n* = 10) [21–23, 33, 47, 50, 58, 60, 61, 69], unable to complete an outcome measurement (*n* = 1) [44] and participants’ co-morbidities might influence the results (*n* = 3) [39, 62, 68]. Five studies provided justification details on why participants were excluded based on the type of medication [33, 38, 57, 62, 68].

#### Potential participant exclusion counts and proportions

Seven studies provided counts of potential participants excluded [29, 30, 52, 55, 59, 60, 67]. Two studies excluded 243 of 791 potential participants because they lived outside the catchment area [30, 59].

Of the 46 studies [21–31, 33–41, 44–48, 50–70] that excluded participants owing to disability (Table 2), only five studies [52, 55, 59, 60, 67] provided counts of potential participants, as follows: 3 of 103 potential participants were excluded because they required assistive devices [52]; 3 of 133 potential participants were excluded owing to cognitive/visual impairments; and 5 of 133 potential participants were excluded because they used limb prosthetics [60]. Eleven of 233 potential participants were excluded owing to the severity of their disability (Steinbocker functional class IV); 39 of 233 potential participants were excluded owing to the presence of other autoimmune diseases and 28 of 233 owing to contraindications to exercise [55]. Two studies excluded 18 of 310 potential participants owing to acute/chronic co-morbidities [55, 67]. One study excluded 2 of 391 potential participants owing to hospitalization [59]. One study excluded 10 participants out of 281 owing to malignancy, intestinal perforation, manic episode and substance abuse [29]. Two studies excluded 26 of 414 potential participants owing to cardiovascular conditions [29, 60]. Three studies reported exclusion of 9 of 571 potential participants owing to recent/planned surgery [52, 59, 67]. One study reported exclusion of 21 of 391 potential participants owing to drug treatment [59]. One study excluded 11 of 103 potential participants because they were already taking part in regular exercise [52], and one study excluded 17 of 77 potential participants owing to sleep and pain issues [67]. One study excluded 11 of 233 potential participants owing to medication [55].

#### Baseline characteristics

At least one PROGRESS-Plus factor was reported in the baseline participants in all 50 studies (Fig. 2; Tables 1 and 2). Religion was the only PROGRESS-Plus factor not reported.

#### Subgroup analysis

PROGRESS-Plus factors were investigated in subgroup analyses in three trials [21, 43, 46]. One study reported a differential effect of exercise on inflammatory markers based on sex (females *vs* males); in this trial, females had reduced inflammatory markers compared with males after the exercise intervention [21]. Another study reported no difference in hand function after an exercise intervention between participants with a disease duration of <5 years compared with ≥5 years or various baseline drug regimens [46]. One study reported that functional capacity and disability were greater after exercise in employed participants compared with participants who retired during the study follow-up period [43].

## Discussion

In this systematic review, we have described the extent to which equity factors were considered within the eligibility criteria, baseline characteristics and subgroup analysis of RCTs evaluating the efficacy of exercise-based interventions for people with RA. All included trials had either some concerns or high risk of bias and reported at least one PROGRESS-Plus equity factor within the eligibility criteria and baseline characteristics. These included place of residence, personal characteristics (age and disability), language, sex, social capital, time-dependent factors and features of relationship factors. No studies excluded participants owing to occupation, religion, education and socioeconomic status. When reported, a total of 457 from 1337 potential participants (34.2%) were excluded based on an equity factor. Of the 457 participants excluded, 243 were owing to place of residence, 162 owing to disability factors, 32 owing to features of relationships and 20 owing to time-dependent factors.

Eligibility criteria are often not justified in published manuscripts owing to word limits. The rationale for excluding adults with RA from participating in exercise-based interventions is often unclear. It might be that exclusions are attributable to the perceived potential for benefit, the target population or the feasibility of participation.

### Perceived potential for benefit

In this review, 46 studies (92%) excluded potential participants based on disability or co-morbidities, particularly cardiovascular conditions. Some studies excluded people with uncontrolled cardiovascular conditions, such as unstable hypertension, the presence of cardiac conditions (e.g. angina, arrhythmia) and recent myocardial infarctions. Excluding potential participants based on unstable or acute cardiovascular conditions might be appropriate owing to the potential for harm. However, other trials excluded participants with common long-term or stable cardiovascular conditions, such as hypertension and chronic heart failure. Although justification for these exclusions was seldom provided, they might be related to an increased risk of myocardial infarction or coronary death for adults with RA when compared with the general population [71].

The prevalence of cardiovascular events in people with RA is declining because of advancements in drug therapy [72], and there is evidence that demonstrates the benefits of exercise for individuals with stable cardiovascular disease and other co morbidities [73, 74]. Consequently, exclusions based on the increased risk of adverse events in people with stable cardiovascular disease might be unjustified and inequitable. From the current review, Lange *et al*. [47] examined the effects of a 20-week personalized moderate- to high-intensity aerobic and resistance programme compared with a low-intensity home exercise programme in older adults (65 years old) with RA. Their study appropriately excluded people with unstable cardiovascular conditions (unstable ischaemic heart disease or arrhythmia) that might preclude participation in moderate-intensity exercises but included participants with stable cardiovascular conditions [47]. The only adverse events reported were attributable to generalized pain, which resolved after reducing exercise for 1 week. No cardiac-related adverse events occurred, and participants exhibited greater aerobic capacity, muscle strength and endurance [47]. This highlights that older adults with stable cardiovascular conditions and RA have potential to benefit from participation in exercise programmes, including interventions being investigated in trials, if given the opportunity. Carefully prescribed and monitored exercise interventions are safe in people with RA; therefore, exclusion based on exercise safety should be minimized, where possible, or justification for exclusions provided.

### Target population

#### Age

Trials of exercise-based interventions define homogeneous populations to reduce variance and the sample size needed. For example, in the trials included in this review, the majority of participants were middle-aged, and nearly half of the RCTs excluded older adults >60 years of age. Some trial designs specifically recruited a target population defined by age or life stage, such as premenopausal women [57] or postmenopausal women [44], to answer their research question. Focusing on these subgroups might be justified because the peak age of RA onset is middle age [75], and identifying appropriate management in this population might minimize disability, health-care costs and work absence [76, 77]. Where the research does not target a specific age group, excluding older adults might not be justified, and people of all ages should be included in order that the findings can be generalized to everyone with RA.

#### Late-onset disease

It is important to include older people with RA in exercise trials because large joint disease contributes to substantial disability in people with late-onset RA [78]. Identifying effective exercise interventions in this subgroup of people with RA is crucial to optimize management. Interestingly, some trials performed more recently addressed this challenge and included only older adults [21, 22, 47]. For example, Anvar *et al.* [22] included female participants aged 60–87 years old, and Andersson *et al.* [21] included participants >65 years old. Exercise in these older adults with RA was found to be safe [21, 22, 47] and improved aerobic capacity [21], muscle strength [21], inflammatory markers [21] and self-efficacy [22]. Furthermore, older adults with RA who participated in moderate- to high-intensity exercise programmes maintained significantly higher physical activity levels at 12 months compared with an age-matched population who participated in a home-based low-intensity exercise programme [47]. Given that physical activity levels tend to be low in older adults and people with RA [79], exercise interventions could provide a wide range of health benefits among older adults with RA. Indeed, trial designs should optimize accessibility and acceptability to maximize participation and ensure that the potential health benefits of exercise are available to everyone.

### Feasibility of participation

#### Language

Another potential reason for excluding people from trials might be the feasibility of participation. In the present review, participants were excluded because they could not speak the native language, and there was the potential for misunderstanding the trial processes and non-adherence to the intervention [21, 33]. There was a lack of funding for translators, and alternative solutions to facilitate the inclusion of non-native language speakers were not considered. Researchers should maximize participation by providing translators where possible. However, these options might not be available, and eligibility might be limited to meet time and funding restrictions. Given that RCTs are often publicly funded, if time and funding constraints limit the generalizability of a trial, the potential cost–benefit of conducting the trial at all should be questioned.

#### Cognitive impairment

In this review, RCTs excluded participants with cognitive impairment owing to concerns regarding capacity to consent and their ability to participate effectively in the study [21–23, 33, 47, 50, 58, 60, 61, 70]. However, people with mild cognitive impairments (including dementia) can adhere to strengthening and endurance-based exercise-based with appropriate adaptations [80]. The RCTs within this review did not specify the level of cognitive impairment that resulted in exclusion and did not provide solutions to overcome this exclusion, such as using carers, memory books or adapting intervention delivery. Consequently, these vulnerable populations were denied access to exercise trials that might improve their health outcomes.

The Marmot Report [6] recommended the use of health equity filters within health-based research and guidelines to identify avoidable health inequities. More recently, the National Institute for Health Research [81] published guidance to address the inclusion of underrepresented groups, such as non-native language speakers or people with cognitive impairment, within clinical research. Systematically excluding those who are likely to incur the greatest health-care costs will fail to generate the health economic evidence base required to change health-care funding for these individuals. Collaborative decision-making between researchers and key stakeholders throughout the research process might also help to identify inequitable practice and feasible solutions to facilitate participation from underserved groups [81].

### Methodological considerations

Firstly, to our knowledge, this was the first systematic review to have used an established health equity framework to identify potential inequity within exercise-based trials for people with RA. Secondly, the protocol for this study was registered on PROSPERO to ensure transparency of our objectives and review methods. Thirdly, the search strategy included published, unpublished and ongoing trials. Finally, screening, selection and quality appraisal were completed in duplicate. However, data extraction was completed by one reviewer and checked for accuracy by a second reviewer; this might have led to some errors in extraction. Furthermore, trials not published in the English language and those published before 2000 were excluded, which might have led to the exclusion of potentially relevant RCTs and an underestimation of the extent to which equity factors were considered by RCTs of exercise interventions for adults with RA.

### Conclusion

This review identified the exclusion of potential participants within exercise-based interventions for people with RA based on equity factors that might affect health-care opportunities and outcomes. It is crucial that participation in exercise-based trials is optimized, because this evidence is used to inform management and service design. Where exclusion criteria are applied, an evidence-informed justification or reasons why participation could not be supported should be stated. All people with RA should be offered an equitable opportunity to improve their health, including participation in research design and delivery, where possible.

## Supplementary Material

rkac095_Supplementary_Data

## Data Availability

The data underlying this article are sourced from the public domain and are available in the articles cited throughout.
