# Supplementary material for: Inequity in exercise-based interventions for adults with rheumatoid arthritis: a systematic review
Source: Rheumatol Adv Pract. 2023 Jan 24;7(1):rkac095. doi: 10.1093/rap/rkac095 (PMC9880983; doi:10.1093/rap/rkac095)
Supplement: rkac095_Supplementary_Data [file rkac095_supplementary_data.zip › rkac095_Supplementary_Data/Supplementary Data S1. Search strategies.docx]

**Supplementary Data S1. Database search strategies**

Embase Search Strategy via OVID: (7824 Studies)

1) exp exercise/

2) (exercis* or aerobic* or cardiovascular* or Walk* or endurance* or Physical activit*).mp. [mp=title, abstract, heading word, drug trade name, original title, device manufacturer, drug manufacturer, device trade name, keyword, floating subheading word, candidate term word]

3) (strength* or resistance*).mp. [mp=title, abstract, heading word, drug trade name, original title, device manufacturer, drug manufacturer, device trade name, keyword, floating subheading word, candidate term word]

4) (aqua* or hydrotherap* or swim* or yoga* or tai chi*).mp. [mp=title, abstract, heading word, drug trade name, original title, device manufacturer, drug manufacturer, device trade name, keyword, floating subheading word, candidate term word]

5) (Flexib* or stretch* or range motion or movement).mp. [mp=title, abstract, heading word, drug trade name, original title, device manufacturer, drug manufacturer, device trade name, keyword, floating subheading word, candidate term word]

6) (balance* or proprio*).mp. [mp=title, abstract, heading word, drug trade name, original title, device manufacturer, drug manufacturer, device trade name, keyword, floating subheading word, candidate term word]

7) exp rheumatoid arthritis/

8) (Rheumatoid arthriti* or ra or rheumatoid nodule* or felty* syndrome or caplan* syndrome or Sjogren* syndrome or still* disease).mp. [mp=title, abstract, heading word, drug trade name, original title, device manufacturer, drug manufacturer, device trade name, keyword, floating subheading word, candidate term word]

9) exp randomized controlled trial/

10) (randomized controlled trial or clinical trial* or rct).mp. [mp=title, abstract, heading word, drug trade name, original title, device manufacturer, drug manufacturer, device trade name, keyword, floating subheading word, candidate term word]

11) 1 or 2 or 3 or 4 or 5 or 6

12) 7 or 8

13) 9 or 10

14) 11 and 12 and 13

Search Strategy for Medline via OVID: (2429 Studies)

1. Exp Exercise/
2. (Exercise* or aerobic* or cardiovascular* or walk* or endurance* or physical activit*).mp.
3. (strength* or resistance*).mp.
4. (aqua* or hydrotherapy* or swim* or yoga* or tai chi* or flexib* or stretch* or range of motion or movement* or balance* or proprio*).mp.
5. Exp rheumatoid arthritis/
6. (rheumatoid arthritis or ra or rheumatoid nodule* or felty* syndrome or caplan* syndrome or Sjogren* syndrome or still* disease).mp.
7. Exp randomized controlled trial/
8. (randomized controlled trial or randomi?ed controlled trial* or clinical trial* or rct).mp.
9. 1 and 2 and 3 and 4
10. 5 and 6
11. 7 and 8
12. 9 and 10 and 11

CINAHL Search Strategy: (324 studies)

1. (MH “Exercise+”)
2. Exercise*
3. Physical activit*
4. Aerobic*
5. Cycling
6. Cardiovascular*
7. Walk*
8. Endurance*
9. Strength*
10. Resistance*
11. Flexib*
12. Stretch*
13. “range of motion”
14. Movement*
15. Hydrotherapy*
16. Aquatic*
17. Swim*
18. Balance*
19. Propriocep*
20. Yoga*
21. Tai chi*
22. S1 OR S2 OR S3 OR S4 OR S5 OR S6 OR S7 OR S8 OR S9 OR S10 OR S11 OR S12 OR S13 OR S14 OR S15 OR S16 OR S17 OR S18 OR S19 OR S20 OR S21
23. (MH “Arthritis, Rheumatoid”)
24. Rheumatoid nodule*
25. Felty* syndrome
26. Caplan syndrome*
27. Sjogren* syndrome
28. Still* disease
29. S23 OR S24 OR S25 OR S26 OR S27 OR S28
30. (MH “Randomized Controlled Trials”)
31. Randomi?ed control
32. Control clinical trial*
33. Clinical trial*
34. Rct
35. S30 OR S31 OR S32 OR S33 OR S34
36. S22 and S29 AND S35

PEDro Search Strategy – (315 studies)

PEDro Search Strategy (available at pedro.org.au) - 127 results

- Abstract & title: Exercis* AND Rheumatoid Arthritis
- Method: clinical trial

PEDro Search Strategy (available at pedro.org.au) - 75 results

- Abstract & title: Rheumatoid Arthritis
- Therapy: Fitness Training
- Method: clinical trial

PEDro Search Strategy (available at pedro.org.au) - 28 results

- Abstract & title: Rheumatoid Arthritis
- Therapy: Hydrotherapy , balneotherapy
- Method: clinical trial

PEDro Search Strategy (available at pedro.org.au) - 85 results

- Abstract & title: Rheumatoid Arthritis
- Therapy: Strength training
- Method: clinical trial

OpenGrey – 11 results

Exercis* AND rheumatoid arthritis

ISRCTN Registry – 14 results

rheumatoid arthritis AND exercise

*Categories selected were: (condition) and (intervention)*
