# Supplementary material for: Inequity in exercise-based interventions for adults with rheumatoid arthritis: a systematic review
Source: Rheumatol Adv Pract. 2023 Jan 24;7(1):rkac095. doi: 10.1093/rap/rkac095 (PMC9880983; doi:10.1093/rap/rkac095)
Supplement: rkac095_Supplementary_Data [file rkac095_supplementary_data.zip › rkac095_Supplementary_Data/Supplementary Table S1. Risk of bias assessment.docx]

| **Supplementary Table S1: Risk of Bias Assessment** | | | | | | |
| --- | --- | --- | --- | --- | --- | --- |
|  | **Domain 1: Randomisation Process** | **Domain 2: Deviations from Intended Interventions** | **Domain 3: Missing Outcome Data** | **Domain 4: Measurement of the Outcome** | **Domain 5: Selection of the Reported Result** | **Overall Judgement** |
| Anvar, 2018 |  |  |  |  |  |  |
| Andersson, 2020 |  |  |  |  |  |  |
| Azeez, 2020 |  |  |  |  |  |  |
| Baillet, 2009 |  |  |  |  |  |  |
| Bearne, 2002 |  |  |  |  |  |  |
| Bilberg, 2005 |  |  |  |  |  |  |
| Breedland 2011 |  |  |  |  |  |  |
| DaSilva, 2013 |  |  |  |  |  |  |
| Dejong, 2004 |  |  |  |  |  |  |
| Durcan, 2014 |  |  |  |  |  |  |
| Ellegaard, 2019 |  |  |  |  |  |  |
| Eurenius, 2008 |  |  |  |  |  |  |
| Eversden, 2007 |  |  |  |  |  |  |
| Feldthusen, 2016 |  |  |  |  |  |  |
| Figen, 2011 |  |  |  |  |  |  |
| Flint-Wagner, 2009 |  |  |  |  |  |  |
| Ganesan, 2020 |  |  |  |  |  |  |
| Garcia-Morales, 2020 |  |  |  |  |  |  |
| Gautam, 2020 |  |  |  |  |  |  |
| Gautam, 2021 |  |  |  |  |  |  |
| Gautam, 2019 |  |  |  |  |  |  |
| Hakkinen, 2004 |  |  |  |  |  |  |
| Hakkinen, 2003 |  |  |  |  |  |  |
| Hale, 2016 |  |  |  |  |  |  |
| Hsieh, 2009 |  |  |  |  |  |  |
| Jahanbin, 2014 |  |  |  |  |  |  |
| Lamb, 2015 |  |  |  |  |  |  |
| Lange, 2019 |  |  |  |  |  |  |
| Lemmey, 2009 |  |  |  |  |  |  |
| Lineker, 2001 |  |  |  |  |  |  |
| Lourenzi, 2017 |  |  |  |  |  |  |
| McKenna, 2021 |  |  |  |  |  |  |
| Mohanty, 2018 |  |  |  |  |  |  |
| Moonaz, 2015 |  |  |  |  |  |  |
| Munneke, 2003 |  |  |  |  |  |  |
| O'Brien, 2006 |  |  |  |  |  |  |
| Ogata-Medal, 2018 |  |  |  |  |  |  |
| Puksic, 2021 |  |  |  |  |  |  |
| **Author and Year** | **Domain 1: Randomisation Process** | **Domain 2: Deviations from Intended Interventions** | **Domain 3: Missing Outcome Data** | **Domain 4: Measurement of the Outcome** | **Domain 5: Selection of the Reported Result** | **Overall Judgement** |
| Rezaei, 2020 |  |  |  |  |  |  |
| Rodriques, 2020 |  |  |  |  |  |  |
| Seneca, 2015 |  |  |  |  |  |  |
| Siqueira, 2017 |  |  |  |  |  |  |
| Strasser, 2011 |  |  |  |  |  |  |
| Surabhi, 2018 |  |  |  |  |  |  |
| Van Rensburg, 2012 |  |  |  |  |  |  |
| Vandenberg, 2006 |  |  |  |  |  |  |
| Veldhunijzen, 2021 |  |  |  |  |  |  |
| Wang, 2008 |  |  |  |  |  |  |
| Ward, 2014 |  |  |  |  |  |  |
| Westby, 2000 |  |  |  |  |  |  |
